# Beyond Journals—Visual Abstracts Promote Wider Suicide Prevention Research Dissemination and Engagement: A Randomized Crossover Trial

**DOI:** 10.3389/frma.2020.564193

**Published:** 2020-10-14

**Authors:** Adam S. Hoffberg, Joe Huggins, Audrey Cobb, Jeri E. Forster, Nazanin Bahraini

**Affiliations:** ^1^Rocky Mountain Mental Illness Research, Education and Clinical Center, Department of Veterans Affairs, Eastern Colorado Health Care System, Aurora, CO, United States; ^2^Department of Physical Medicine and Rehabilitation, University of Colorado School of Medicine, Aurora, CO, United States; ^3^Department of Psychiatry, University of Colorado School of Medicine, Aurora, CO, United States

**Keywords:** veterans, social media, suicide prevention, twitter, open science, altmetric, randomized crossover design

## Abstract

**Background:** Many academic institutions and journals disseminate research through social media to increase accessibility and reach a wider audience. “Visual Abstracts” are well-suited for social media dissemination, and have been adopted by some as a novel approach to increase engagement with academic content. Visual abstracts are a visual representation of key methods and findings from a traditional peer-reviewed publication. This study expands on previous research by examining the impact of visual abstracts compared to traditional text abstracts to disseminate research produced in a national research center focused on preventing Veteran suicide.

**Methods:** A prospective, randomized crossover design was utilized to compare Twitter posts with a visual abstract to those with a simple screen grab of the PubMed abstract (*n* = 50 journal publications). Outcomes were measured using native Twitter Analytics to track impressions, retweets, total engagements, and link clicks about 28 days post-tweet, and Altmetric It to track additional alternative metric outcomes.

**Results:** Visual abstract tweets were associated with a significantly higher number of impressions (*p* < 0.001), retweets (*p* < 0.001), and link clicks (*p* = 0.02) compared with text abstract tweets.

**Conclusions:** In line with results from prior studies, we found that visual abstracts resulted in significantly greater research dissemination and social media engagement via retweets and link clicks compared with text tweets. These findings provide further evidence that visual abstracts increase awareness and readership of journal publications, and that Twitter is an effective platform for research dissemination beyond the traditional academic researcher audience. Implications highlight the importance of social media for suicide prevention advocates, Veteran health researchers and other stakeholders to communicate research findings.

## Introduction

It has been 20 years since Balas and Boren (2000) stated that 86% of research findings never come to be used in health care practice and the 14% that do make it to practice will take 17 years to arrive (Balas and Boren, 2000). This is widely known as the research to practice gap. For research to have any chance of being translated into clinical practice, it must come to the attention of its intended audience (e.g., policy makers, healthcare providers, and healthcare consumers). Even the most robust research finding with clear clinical implications will have relatively little value in the world of science and medicine if it is never read. Though the importance of dissemination is widely accepted, we explore three overarching barriers that keep scientific findings buried in journals—unread, unappreciated, and ultimately unhelpful to society.

The first obstacle is the issue of readability. Plaven-Sigray et al. (2017) analyzed the readability of over 700,000 abstracts from 1881 to 2015 (Plaven-Sigray et al., 2017). They found that the readability of scientific writing is steadily decreasing, and they also posit that lower readability implies less accessibility to science, particularly for non-specialists, such as journalists, policy-makers and the wider community of stakeholders (Plaven-Sigray et al., 2017). It's easy to see that publications with low readability may cloak important findings behind difficult to comprehend academic jargon. Tim Radford, in an article published in 2011 by *Nature*, stated that “the language, form and conventions of the published scientific paper could almost have been devised to conceal information,” using words that the general public will have never heard or used (p. 445) (Radford, 2011). To remedy this, he suggests that scientists step back and view their work from other perspectives (Radford, 2011).

Second, there are important limitations of more traditional print-based distribution methods. Held captive behind expensive paywalls, many publications are simply not able to be accessed by the stakeholders that rely on the research to make evidence-informed health care decisions. Open access (OA) refers to freely available scholarly literature. The OA movement pushes for more research publication content that is easy to find and use. With growing interest in the rapid dissemination of science, OA plows a wider path to research accessibility. In fact, a large-scale study of over 67 million articles assessing the prevalence and characteristics of OA found that as of 2015, at least 28% of publications are OA (about 19 million articles), and that this proportion is growing (Piwowar et al., 2018). Furthermore, OA has led to increased uptake of research. The researchers found that OA articles receive 18% more citations than average (Piwowar et al., 2018). The momentum and possibilities of OA impact is accelerating with the evolution of the internet. A 2008 study confirmed that OA nearly doubled the likelihood that mental health professionals would read relevant articles if they are freely available online (Hardisty and Haaga, 2008). However, so called “access tolls” remain a hindrance to the dissemination and implementation of research published within even the most prestigious academic journals.

The sheer volume of publications is a third daunting obstacle. The Scientific, Technical and Medical (STM) Report 2018 from the International Association of Scientific, Technical and Medical Publishers estimated that in 2018 there were three million articles published (Johnson et al., 2018). With so many published works, the onus is often on the reader to parse out what is worth attention. Even articles with interesting findings and implications can be overlooked in the growing sea of scientific literature (Bornmann and Mutz, 2015). This exponential growth leads to unmanageable amounts of information. Even if research overcomes the first two barriers and is both readable and accessible, it seems insurmountable for stakeholders to keep up with advances *in the traditional text form*.

### Rapid Dissemination in a Digital World

While these obstacles are formidable, opportunities exist to make published work stand out, and the internet has indisputably changed the way researchers and organizations disseminate information. A paradigm shift in science means publications are not the endpoint, merely a point along the continuum of research communication. Researchers are mobilizing their digital presence to boost the rapid dissemination of their work and explore new opportunities to reach their peers and the wider community of stakeholders. A digital presence opens the possibilities of discoverability; therefore, many academic, scientific, governmental, health, and journal organizations have pivoted to social media as a revolutionary tool to disseminate research, increase accessibility, and reach a wider audience. Social media enables the immediate exchange of information and ideas and promises to transform how research is communicated and translated into healthcare practices.

Social media consists of many different platforms to serve diverse needs, and Twitter in particular has evolved into a central online hub for lifelong learning (Kind and Evans, 2015). Twitter is a microblogging social media outlet that allows users to post messages up to 280 characters in length. Surveys of researchers found that ~10–15% used microblogging tools (Rowlands et al., 2011; Grande et al., 2014), and Twitter has emerged as the premiere microblogging tool in scholarly communication. Twitter is regularly used to announce new journal issues, promote individual articles, and engage with readers. Given the limited text length requirements, social media also unlocks the prospect of presenting key information from studies in a condensed, digestible format. For example, social posts can serve as a sounding board for discussing research and its implications for preventing suicide among a wide variety of stakeholders. In addition to academic researchers, Twitter garners widespread utilization among mental health professionals, as well as those with lived experience, such as individuals who have survived a suicide crisis and those who have lost a loved one to suicide. Prior work has also shown that Twitter's impact correlates with traditional citation impact (e.g., frequently tweeted articles go on to have more citations) (Eysenbach, 2011).

### Rise of Visual Abstracts

“Visual Abstracts” offer a promising solution to address readability, accessibility, and draw attention to significant research. Visual abstracts are an emerging social media dissemination approach, defined as a visual representation of the key methods and findings from a traditional journal publication^1^. The visual abstract is a subset of the graphical abstract, which first found use in the mid-1970's in chemistry journals (2011). Graphical abstracts have been shown to increase performance of manuscripts in terms of downloads, views, and citations (Pferschy-Wenzig et al., 2016).

The goal of the visual abstract is to present information in a compelling visual way that lets the viewer decide whether to pursue “the rest of the story” found within the scientific journal publication. At its heart, a visual abstract is intended to reflect the earnest desire to disseminate and share scientific knowledge^1^. Ibrahim et al. pioneered the modern day visual abstract format^1^. They outline the following guidelines and design principles when developing a visual abstract: focus on the user experience, clear purpose/focus, rapid prototyping/iterative development, thoughtful restraint, and relevant creativity^1^. In essence, visual abstracts are an attempt to make scientific content more accessible, without compromising message quality. Creating a visual abstract requires distilling concepts down to only their most important details.

Prior studies showed significant positive effects of visual abstracts to increase engagement within specific academic fields [e.g., surgery (Ibrahim et al., 2017; Chapman et al., 2019), geriatrics (Lindquist and Ramirez-Zohfeld, 2019)]. Research designs to test their impact include retrospective cross-sectional evaluation (Koo et al., 2019), as well as more rigorous randomized prospective approaches (Ibrahim et al., 2017; Chapman et al., 2019). In the landmark study testing visual abstracts, Ibrahim et al. (2017) conducted a prospective case-control crossover study of *Annals of Surgery* publications (Ibrahim et al., 2017). This journal is the world's most referenced surgery journal, and they found a strong correlation between the use of visual abstract tweets and increased dissemination on social media (Ibrahim et al., 2017).

Although visual abstracts originated in the field of surgery in July 2016, they have since been adopted as a novel approach by a growing body of institutions into routine journal practices^1^. Diverse disciplines utilizing visual abstracts include nephrology (Colbert et al., 2018), venous and lymphatic (Gloviczki and Lawrence, 2018a), vascular, rectal, and head/neck surgery (Nikolian and Ibrahim, 2017; Gloviczki and Lawrence, 2018b; Villwock and Johns, 2018), transplantation (Henderson et al., 2019), gastroenterology (Ibrahim, 2018), urology (Koo et al., 2019), and cardiovascular (Ibrahim and Bradley, 2017) research. Perhaps most notably, the New England Journal of Medicine regularly incorporates visual abstracts into Tweets about new scholarly publications^2^.

However, widespread implementation remains limited to specialized fields of science, and is particularly nascent in mental health. While webinars are available with anecdotal reports regarding the use of visual abstracts in Veterans health research domains (Connelly and Gilmartin, 2019), the authors are not aware of any published research that evaluated visual abstracts in the realms of both Veterans and mental health/suicide prevention.

### Aims of the Current Study

In the fall of 1997, Congress commissioned the Department of Veterans Affairs (VA) to establish Mental Illness Research, Education and Clinical Centers (MIRECC) with the goal to “decrease the time it takes clinical best practices to move from the literature to daily clinical practice (p. 119)”(Bryan et al., 2019). The Rocky Mountain MIRECC was established in 2004, and part of its mission is to disseminate useful information about suicide prevention in ways that are accessible to Veterans and the community at large, as well as evaluate strategies to translate research-informed practices into everyday care (Bryan et al., 2019).

Given the sustained rise in suicide rates among both Veterans and non-Veterans in the U.S. over recent decades (Hedegaard et al., 2020), innovations in suicide prevention are more urgent than ever. Consequently, this study was undertaken to evaluate the extent to which a Twitter dissemination strategy using visual abstracts influences outcomes on awareness and readership of Rocky Mountain MIRECC journal publications covering Veterans' mental health, suicide prevention, and related topics. Suicide prevention is particularly ripe for the implementation of novel dissemination tactics, as it is imperative that a broad range of stakeholders both within and outside academia remain current on research that advances best practices.

The current study tests a strategy to reach a wider audience in suicide prevention research, and extends the limited body of literature to help organizations, including the VA, understand the potential impact of implementing visual abstracts into research communication. While prior research has focused on creating and disseminating visual abstracts for specific journal content, there are some key distinctions this study adds to the literature. First, this study covered published research spanning many journals. Specifically, Rocky Mountain MIRECC publications represent multidisciplinary topic areas and audience interests, including public health, neuroscience, rehabilitation, psychology, social work, counseling, and microbiology, among others. These audience segments differ from the more homogenous audience in previous studies (e.g., Surgery). Furthermore, unlike academic journals, healthcare systems and organizations have a direct line to providers and patients and therefore are uniquely positioned to engage a broader group of stakeholders than those who normally subscribe to academic journals. In fact, large portions of suicide prevention audiences (e.g., individuals with lived experience) do not subscribe to medical journals. By extending this strategy to healthcare organizations who interact with patients, families, providers, advocates, and policy makers, we sought to communicate timely research outputs from our center in a public and accessible way. Moreover, this effort is part of a larger strategy focused on using social media to communicate and raise awareness about Veteran's mental health research topics and resources to prevent suicide.

The aim of this study is to test the effects of incorporating visual abstracts into Rocky Mountain MIRECC social media dissemination efforts. We expand on previous work by outlining a reproducible approach including examples and self-guided training that could be adopted by other researchers and organizations in which dissemination and timely communication of research findings is a key part of their mission.

### Research Questions

The study research questions were (see Supplemental Table 1 for definitions): Compared with text abstract tweets, are visual abstract tweets associated with an increased number of times the:

- Tweet is seen (impressions^3^–primary outcome)?
- Tweet is shared (retweets^3^–secondary outcome)?
- Article link is clicked (link clicks^3^–secondary outcome)?

In addition, we aimed to examine how visual and text abstract tweets impact alternative metrics attention scores (Altmetric^4^– outcome). In *post-hoc* analyses, retweets were assessed to identify engagement and reach to practitioners and others on Twitter such as those with lived experience in the suicide prevention community.

## Methods

This research was conducted and reported in accordance with the Consolidated Standards of Reporting Trials (CONSORT) extension to randomized crossover trials (Dwan et al., 2019). A completed CONSORT checklist is available (see Supplemental Table 5).

### Ethics Approval

This study was reviewed and approved by the Colorado Multiple Institutional Review Board (COMIRB) and by the VA Eastern Colorado Healthcare System (ECHCS) Research and Development ethics committee. The protocol was determined to be not human subjects research and therefore exempt from clinical trial registration.

### Study Design

**Figure 1:**
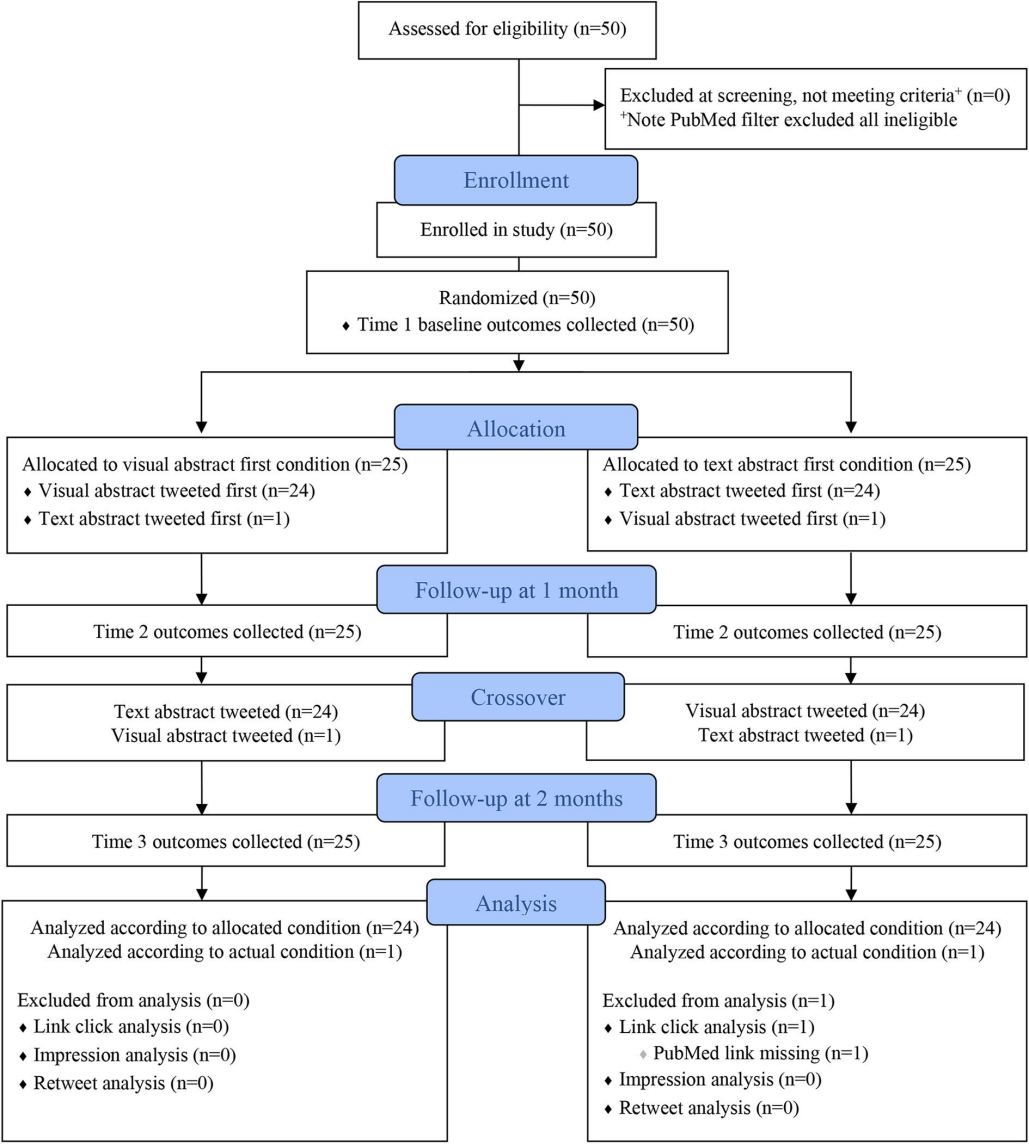
Consolidated Standards of Reporting Trials (CONSORT) Extension to Crossover Trials Flow Diagram.

A prospective, randomized two-period crossover trial was conducted to randomize (*n* = 50) journal publications comparing Twitter posts with a visual abstract to those with a text abstract, defined as a simple screen grab of the PubMed abstract. Publications were block randomized, with a 1:1 allocation ratio, to either the visual abstract first condition, or the text abstract first condition, followed by a 28-day washout period and crossover to the other condition (see Figure 1). This extended washout period limits any crossover contamination effects and the length is consistent with Ibrahim et al. (2017) and research suggesting that the average half-life of a tweet is only 24 minutes^5^. The randomization scheme contained random block sizes and was created by the study biostatistician (JF) using PROC PLAN in SAS v9.4. For the fourth publication randomized, an error was made such that the visual abstract was tweeted first but the publication was in the text first condition. Upon discovery, the next publication randomized to the visual first condition was changed to the text first condition to maintain balance by the end of the study.

### Eligibility Criteria

Articles were included if the publication was indexed in PubMed, had at least one author with a Rocky Mountain MIRECC affiliation, and was published on or after June 1, 2018 according to the PubMed Published Date or Create Date. Publications were excluded if there was no full text access available or if the Rocky Mountain MIRECC @RMIRECC Twitter account had previously posted about them prior to study commencement. Publications were sequentially enrolled from a custom PubMed alert that searched for all known Rocky Mountain MIRECC investigators. Due to the criteria informing the PubMed query, all studies that returned in the alert met eligibility criteria and were sequentially enrolled. Study enrollment commenced June 1, 2018 and concluded April 4, 2019 when the recruitment goal was met (*n* = 50) (see Supplemental Table 2 for all included publications).

### Visual Abstract Creation

Following publication enrollment, articles were assigned to a member of the research team for visual abstract creation. There is not enough space on a visual abstract to write complex sentences, and ideas were translated to be conveyed visually as much as possible. Efforts were made to reduce overly scientific or technical language, and most acronyms were defined on the canvas. Each visual abstract went through an interactive review process among the research team that culminated in consensus and final approval by the senior member (NB). All visuals were reviewed by the study team before they were complete, which provided an opportunity to see the work from another's perspective and ensure coherence. The visual was informed by the publication itself and efforts were made to not consult with authors from the enrolled study. Standardized components of a visual abstract include summary and display of key questions and outcomes, citation, and creator. Examples of the highest performing visual abstracts from this study are available (see Supplemental Table 3).

### Study Tweet Procedures

All study tweets from both conditions were required to post from the @RMIRECC Twitter account^6^ according to a standardized procedure. In order to reduce the risk of bias due to confounding, all tweets included the exact title of the article, and no additional hashtags were used (e.g., we did not use #VisualAbstract), nor were potentially relevant Twitter user accounts tagged in the posts. All study tweets were posted in the morning (Mountain Standard Time).

### Outcome Measurement

Once the visual abstract image was approved by the study team, Time 1 baseline outcomes were measured, and then the initial tweet was posted according to the randomized allocation. Following a 28-day washout period (±3 days), Time 2 outcomes were measured, and then every article crossed over and was tweeted in the other condition, such that each article was tweeted twice, once as a visual abstract, and once as a text abstract. After a second 28-day period (±3 days), the final Time 3 outcomes were measured. Each publication was enrolled in the study for ~2 months (see Figure 1).

### Availability of Data and Materials

Publicly available data were collected via the Twitter and Altmetric platforms. Native Twitter Analytics were the outcome measurement source for impressions (primary outcome), retweets, total engagements, and link clicks (secondary outcomes). Altmetric It^7^ was used to measure additional alternative metric outcomes (exploratory outcome). Altmetrics are an “attention score,” providing complementary data indicators of activity in online tools and environments. They count societal impact, broadly measured by mentions in news, social media, blogs, and reference manager readers. The timing of outcome measurement and data sources are described (see Supplemental Table 4).

### Analysis Plan

This study utilized a two-period crossover design. Condition effects were determined using a crossover design specific analysis that assumed no carry-over effects, given the substantial washout period. Additionally, as the outcome measures were found to be highly non-normal, a non-parametric approach was used. All analyses assumed a two-sided test of hypothesis, a significance level of 0.05 and were run in SAS v9.4. Prior to analysis, a Fisher's exact test was used to determine if there was an association between condition allocation and who created the visual abstracts. As this was highly non-significant (*p* = 0.85), this was not considered further. The analysis of treatment effect entails taking one-half of the difference within publication and between periods, with the subtraction order dependent on the sequence (text first vs. visual first). The medians are then compared between the sequences using a Wilcoxon rank-sum test, which tests the effect of the type of tweet (Tudor and Koch, 1994). Data for total engagement was exploratory and therefore only described. Mean and median differences between conditions are presented. *Post-hoc* analyses assessed exploratory retweet outcomes for signals regarding audience reach of study tweets.

## Results

Visual abstract tweets were associated with a significant increase in impressions (median increase = 148; *p* < 0.001), retweets (median increase = 2; *p* < 0.001), and clicks (median increase = 1; *p* = 0.02) as compared to text abstract tweets. Median increases remained the same when the two publications affected by the randomization error were removed from analysis and significance increased slightly for all three tests (data not shown). After it became apparent that some study tweets had not been properly indexed by the Altmetric platform, Altmetric scores were determined to be unreliable, and results are therefore not presented. While not tested, the median difference in total engagements was 6, such that visual abstract tweets had a higher number of engagements. All results are presented (see Table 1).

**Table 1:** Within abstract differences (Visual minus Text) *N* = 50.

	**Mean difference (SD)**	**Median difference (Range)**	**Wilcoxon rank-sum *p*-value**
Impressions	435 (830)	148 (−482, 3949)	0.0004
Retweets	2.18 (3.6)	2 (−6, 14)	0.0002
Link clicks^*^	1.31 (4.7)	1 (−11, 18)	0.02
Engagements	10.1 (20.0)	6 (−29, 78)	n/a

* *n = 49; SD = standard deviation; n/a = not applicable*.

In the exploratory results, we found that study tweets reached practitioners and others outside of the Rocky Mountain MIRECC scientific research community, and preliminary analyses suggested this audience may engage with visual abstract tweets more. Each visual abstract tweet was retweeted by this audience on average 2.08 times compared with 0.82 retweets for text abstract tweets.

## Discussion

This study examined a novel approach to augment the attention of Rocky Mountain MIRECC research publications. Through this randomized crossover design, both social media engagement and reach was boosted using visual abstracts. Thus, significant evidence emerged to support the ongoing implementation of visual abstracts in social media dissemination of Rocky Mountain MIRECC publications. This study tested visual abstracts produced by a government research institution whose investigators publish across a wide range of Veterans, mental health, and suicide prevention research topics catering to a multidisciplinary audience of stakeholders. A unique aspect of this study is that we sought to reach a wider audience and identify signals of engagement by non-researchers.

These positive findings are not surprising in that they reflect our relatively well-characterized affinity to process visual information. Dr. Tufte, an early pioneer in the field of data visualization, found that humans process visual data better and faster than other types of data (Tufte, 1942). Digital marketing strategists in particular have long taken advantage of this preference for visual content to engage with their consumer audience. In their commentary “#VisualAbstract: A Revolution in Communicating Science?” Wray and Arora remind us that webpages with videos and images draw, on average, 94% more views than their text-only counterparts (Wray and Arora, 2017). It is no wonder then that the visual abstract approach is spreading rapidly to many researchers and organizations.

### Practical Considerations for Implementation

Since visual abstracts are relatively low effort, inexpensive, and easily implemented, and with this confirming evidence informing our efforts, the Rocky Mountain MIRECC adopted an ongoing visual abstract dissemination strategy on Twitter. Since adoption, we have published 35 additional visual abstracts to Twitter that were not part of this research study. Lessons learned moving beyond the research study include editorial discussions selecting publications for visual abstracts. The best fit are articles with generally straightforward research questions and findings, terms that don't need acronyms or complex explanation, and content with concrete concepts that translate to relatively easy visuals to complement the findings. There are also important design considerations for visual abstract creators including the selection of complementary color palettes, and applying appropriate contrast, font, and images.

Since the close of this study, we continue to refine optimal ways to present important research aspects and implications in an engaging visual way. Our approach has evolved to include tagging relevant audiences in the tweets, using #VisualAbstract and other hashtags relevant to the published content, as well as repetition of key messaging and design templates to drive home important messaging about suicide prevention across research findings.

To aid implementation by Rocky Mountain MIRECC investigators and support other organizations in this effort, a visual abstract gallery webpage was launched with examples^8^, along with a self-paced, web-based training module that includes a guided “explainer” video for creating visual abstracts^9^. It is hoped that these publicly available resources will increase uptake and promote widespread adoption by others.

### Strengths

The strengths of this study lie in the rigorous and reproducible methodological study design and analysis used to evaluate visual abstract impact. The standardized data measurement approach we utilized provided objective and reliable data collection for all primary and secondary outcomes via publicly available data sources, as well as complete follow-up for all enrolled studies. Additionally, the @RMIRECC Twitter account is officially verified, representing an authoritative government source for research dissemination and suicide prevention information. There are inherent social capital and reputation rewards for performing the useful service of tweeting links to new scientific articles.

The present study also extends previous research by including Altmetric attention scores as exploratory outcomes, although this source of outcome metrics inherited its own limitations described below.

### Limitations

A crucial limitation of this study is its generalizability. The scope of Rocky Mountain MIRECC research and the relatively niche active Twitter followers of the @RMIRECC account do not necessarily extend to other content areas and social media platforms. Confounding also existed in that Rocky Mountain MIRECC investigators and other like-minded researchers engaged with study tweets, thereby contributing to an “echo chamber” in which findings from this study cannot necessarily be generalized to online public engagement. However, there is some evidence to suggest that study tweets did reach outside the traditional academic science researcher audience. Furthermore, due to time zone differences across followers, study tweets may have reached only a limited group of individuals.

While the incorporation of Altmetric data as an exploratory outcome is a strength of this study, it also introduced its own limitations. Although the Altmetric service is supposed to automatically pick up on online attention that uses the PubMed identifier (PMID)^10^, we found that this was not always the case. Consultation with Altmetric support staff resolved the missed study tweets in question so that they were correctly captured retroactively, but no explanation was provided as to why this occurred for some tweets and not others, nor how to prevent this in the future. The inconsistent capturing of study tweets within Altmetric therefore limited the utility and reliability of the Altmetric attention score and ultimately prevented us from drawing any conclusions about the impact of visual abstracts in this domain. It remains muddled if there are better ways to link out to publications [e.g., via the digital object identifier (DOI)] to ensure that the Altmetric application program interface properly matches the mention on Twitter with the unique research output.

Considering the strict eligibility criteria for this study, no editorial stewardship was applied to decide which publication content was a “best fit” for visual abstracts. It must be acknowledged that not all published research translates well into a visual abstract format. Rocky Mountain MIRECC publications enrolled in this evaluation consisted of many study designs, including quantitative, qualitative, mixed methods, as well as reviews, commentaries, and editorials. Members of the research community, healthcare professionals, and the general public may be attracted to specific research topics, and selective approaches to reach a wider audience with more relevant studies are likely more effective. Different visual abstract design approaches and appropriate level of detail may vary depending on the intended audiences.

It is also possible that interactions with study tweets occurred without triggering engagement metrics (e.g., articles may have been navigated to outside of Twitter), therefore it is not possible to measure all Twitter Analytics outcomes with certainty.

Finally, diffusion of visual abstracts also highlights important perils. Many pitfalls exist, including the danger of oversimplification of the visual in contrast to the rigor of the research itself, biases in selecting visual content, and poor-quality crafting of the visual and/or translation of the research. The quality of the visual could impact engagement outcomes, and we had quality controls in place including a process for internal review.

Ibrahim et al. correctly remind us that visual abstracts are only meant to highlight or preview articles and are not a substitute for reading them (Ibrahim et al., 2017). Unfortunately, access to the full publication is not always possible for the Twitter audience since not all Rocky Mountain MIRECC publications enrolled in this study were OA. It is unclear how OA status may have confounded findings by impacting engagement with study tweets. However, the impact of visual abstracts on research engagement and reach may be further realized as efforts to improve access to federally funded research publications (i.e., PubMed Central) are implemented.

### Future Research

Future efforts should include the study of implementation of visual abstracts at scale and refine processes to maximize engagement. Further research exploring alternative metrics as primary outcomes is warranted. Studies should also expand in scope to determine how social media and Twitter in particular can influence the entire cycle of scientific enterprise, from idea development to communication of findings, all the way to implementation into practice and policy implications. It remains undetermined whether visual abstracts as a communication strategy lead to only superficial increases in awareness and engagement metrics, or meaningfully translate into changes in policy and/or clinical practice. That being said, it is likely that multifaceted strategies are more likely to increase awareness and translation into practice.

Future work should characterize how Twitter and alternative metric signals extend into diffusion of knowledge and changing practices. Network analyses could illuminate how research information spreads across social media networks. Furthermore, case studies tracing the path from research publication to practice implementation may shed additional light on bridging the research to practice gap. For example, we highlight a case from this study, during which a landmark suicide prevention research study was published in *JAMA Psychiatry* and enrolled in this study (Stanley et al., 2018). This large-scale cohort comparison found evidence in support of safety planning as a valuable clinical tool for suicide prevention in health care settings. The visual abstract earned 14 retweets, 5 links clicks, and 58 total engagements compared with 3 retweets, 5 links clicks, and 25 total engagements for the text tweet. Within a rapid period after publication, including widespread attention across many platforms online (Altmetric score 610 at end of study period), this study generated a clinical care policy response from the VA to scale up the intervention across facilities. Many factors contributed to this rapid implementation into practice, and the visual abstract was but one communication tool among a multi-pronged “hub and spoke” approach to help promote awareness about the effectiveness of safety planning and build momentum for widespread implementation within the VA.

As more journals and institutions turn to visual abstracts and other novel ways (e.g., podcasts) of communicating the practical implications of research findings, it will be important to examine which strategies maximize reach and impact to diverse stakeholder audiences. It will be interesting to understand how strategies synergize to achieve meaningful change.

This is especially important given the burgeoning challenges in oversaturation of media online, and future studies need to account for an audience with increasingly divided attentional time. Creative mediums such as animated Graphics Interchange Formats (GIF) visual abstracts and more sophisticated animated/whiteboard style videos may be even more fruitful and complementary strategies for the rapid dissemination of scientific research. Future research should explore these mediums.

## Conclusions

In line with results from prior studies, we found that visual abstracts resulted in significantly greater reach and social media engagement via retweets and link clicks when compared with text tweets. These findings provide further evidence that visual abstracts increase awareness and readership of journal publications, and that Twitter is an effective platform for research dissemination. There are important implications highlighting novel ways to use social media as a tool for suicide prevention researchers and other stakeholders in Veterans health research to communicate findings. Visual abstracts are not a replacement for reading a full scientific article, but the format is a compelling option to increase awareness and readability of suicide prevention research. They may provide an important conduit for communicating advances in suicide prevention to a wider audience outside the scientific research community. Carefully navigating the use of visuals must distinguish effective scholarly communication from the more superficial trap of social media marketing. As scientists, we must remember that the dazzle of creative visuals rests upon the foundation of meaningful application and rigorous research content at its core.

The mission of the Rocky Mountain MIRECC is to end Veteran and all suicide. This requires that our stakeholders understand and have access to the best available evidence in support of this mission. Visual abstracts reveal possibilities for the future of scientific communication as we move beyond the journal article alone.

## Data Availability Statement

Publicly available datasets were analyzed in this study. This data can be found here: Twitter and Altmetric platforms.

## Author's Note

A version of this work was previously presented: AH, JH, AC, JF, and NB (poster presentation). Beyond Journals—Using Visual Abstracts to Promote Wider Research Dissemination. 5th Biennial Conference of the Society for Implementation Research Collaboration. 2019 September 13–14; Seattle, WA.

## Author Contributions

AH, JH, and NB contributed to the conception of the study. AH, JH, NB, and JF contributed to the study design. AH, JH, and AC contributed to visual abstract creation. NB contributed to visual abstract feedback and approval. AH and JH contributed to posting study tweets and outcome collection. JF contributed to the analysis. All authors contributed to the interpretation of findings, as well as drafting, and revision of the manuscript. All authors contributed to the article and approved the submitted version.

## Conflict of Interest

The authors declare that the research was conducted in the absence of any commercial or financial relationships that could be construed as a potential conflict of interest.

## Footnotes

^1^Use of a Visual Abstract to Disseminate Scientific Research. Available online at: www.SurgeryRedesign.com/resources.

^2^Visual Abstracts. Available online at: https://www.nejm.org/multimedia/visual-abstracts.

^3^About your activity dashboard. Available online at: https://help.twitter.com/en/managing-your-account/using-the-tweet-activity-dashboard.

^4^What are Altmetrics? Available online at: https://www.altmetric.com/about-altmetrics/what-are-altmetrics/exploratory.

^5^Your tweet half-life is 1 billion times shorter than Carbon-14's. Available online at: http://www.wiselytics.com/blog/tweet-isbillion-time-shorter-than-carbon14/.

^6^Available online at: https://www.twitter.com/rmirecc

^7^Bookmarklet for Researchers. Available online at: https://www.altmetric.com/products/free-tools/bookmarklet/.

^8^Visual Abstract Gallery. Available online at: https://www.mirecc.va.gov/visn19/education/visualabstracts.asp.

^9^Beyond Journals - Creating Visual Abstracts for Wider Research Dissemination. Available online at: https://www.mirecc.va.gov/visn19/education/visual_abstracts/creating/.

^10^How it works. Available online at: https://www.altmetric.com/about-our-data/how-it-works/.
