# Supplementary material for: Beyond Journals—Visual Abstracts Promote Wider Suicide Prevention Research Dissemination and Engagement: A Randomized Crossover Trial
Source: Front Res Metr Anal. 2020 Oct 14;5:564193. doi: 10.3389/frma.2020.564193 (PMC8028397; doi:10.3389/frma.2020.564193)
Supplement: Supplementary file 1 [file Table_1.DOCX]

# Supplemental Table 1. Definitions

| **Term** | **Definition** |
| --- | --- |
| Impressions | Times a user is served a Tweet in timeline or search results |
| Retweets | Times a user retweeted the Tweet |
| Link clicks | Clicks on a URL (Uniform Resource Locator; e.g., web address) in the Tweet |
| Engagements | Total number of times a user interacted with a Tweet. Clicks anywhere on the Tweet, including retweets, replies, follows, likes, links, cards, hashtags, embedded media, username, profile photo, or Tweet expansion |
| Altmetrics | Metrics and qualitative data that are complementary to traditional, citation-based metrics. They can include (but are not limited to) peer reviews on Faculty of 1000, citations on Wikipedia and in public policy documents, discussions on research blogs, mainstream media coverage, bookmarks on reference managers like Mendeley, and mentions on social networks such as Twitter |
