# Supplementary material for: Beyond Journals—Visual Abstracts Promote Wider Suicide Prevention Research Dissemination and Engagement: A Randomized Crossover Trial
Source: Front Res Metr Anal. 2020 Oct 14;5:564193. doi: 10.3389/frma.2020.564193 (PMC8028397; doi:10.3389/frma.2020.564193)
Supplement: Supplementary file 2 [file Table_2.DOCX]

# Supplemental Table 2. Included Rocky Mountain MIRECC Publications (n=50)

| **Article ID** | **Article Citation** | **PubMed Link** |
| --- | --- | --- |
| 1 | Bahraini NH, Hostetter TA, Forster JE, Schneider AL, Brenner LA. A Rasch analysis of the Neurobehavioral Symptom Inventory in a national cohort of Operation Enduring and Iraqi Freedom veterans with mild traumatic brain injury. Psychol Assess. 2018 Jun 7. doi: 10.1037/pas0000555. [Epub ahead of print] PubMed PMID: 29878819. | <https://www.ncbi.nlm.nih.gov/pubmed/29878819> |
| 2 | Peng X, Brenner LA, Mathai AJ, Cook TB, Fuchs D, Postolache N, Groer MW, Pandey JP, Mohyuddin F, Giegling I, Wadhawan A, Hartmann AM, Konte B, Brundin L, Friedl M, Stiller JW, Lowry CA, Rujescu D, Postolache TT. Moderation of the relationship between Toxoplasma gondii seropositivity and trait impulsivity in younger men by the phenylalanine-tyrosine ratio. Psychiatry Res. 2018 Mar 22. pii: S0165-1781(17)30437-7. doi: 10.1016/j.psychres.2018.03.045. [Epub ahead of print] PubMed PMID: 30057257. | <https://www.ncbi.nlm.nih.gov/pubmed/30057257> |
| 3 | Subramaniam P, Rogowska J, DiMuzio J, Lopez-Larson M, McGlade E, Yurgelun-Todd D. Orbitofrontal connectivity is associated with depression and anxiety in marijuana-using adolescents. J Affect Disord. 2018 Jul 3;239:234-241. doi: 10.1016/j.jad.2018.07.002. [Epub ahead of print] PubMed PMID: 30025312. | <https://www.ncbi.nlm.nih.gov/pubmed/30025312> |
| 4 | Mohatt NV, Billera M, Demers N, Monteith LL, Bahraini NH. A menu of options: Resources for preventing veteran suicide in rural communities. Psychol Serv. 2018 Aug;15(3):262-269. doi: 10.1037/ser0000203. PubMed PMID: 30080083. | <https://www.ncbi.nlm.nih.gov/pubmed/30080083> |
| 5 | Stanley B, Brown GK, Brenner LA, Galfalvy HC, Currier GW, Knox KL, Chaudhury SR, Bush AL, Green KL. Comparison of the Safety Planning Intervention With Follow-up vs Usual Care of Suicidal Patients Treated in the Emergency Department. JAMA Psychiatry. 2018 Jul 11. doi: 10.1001/jamapsychiatry.2018.1776. [Epub ahead of print] PubMed PMID: 29998307. | <https://www.ncbi.nlm.nih.gov/pubmed/29998307> |
| 6 | Hwang J, Legarreta M, Bueler CE, DiMuzio J, McGlade E, Lyoo IK, Yurgelun-Todd D. Increased efficiency of brain connectivity networks in veterans with suicide attempts. Neuroimage Clin. 2018 Apr 23;20:318-326. doi: 10.1016/j.nicl.2018.04.021. eCollection 2018. PubMed PMID: 30105203; PubMed Central PMCID: PMC6086217. | <https://www.ncbi.nlm.nih.gov/pubmed/30105203> |
| 7 | Holliday R, Smith NB, Monteith LL. An initial investigation of nonsuicidal self-injury among male and female survivors of military sexual trauma. Psychiatry Res. 2018 Jul 27;268:335-339. doi: 10.1016/j.psychres.2018.07.033. [Epub ahead of print] PubMed PMID: 30096662. | <https://www.ncbi.nlm.nih.gov/pubmed/30096662> |
| 8 | Simonetti JA, Azrael D, Rowhani-Rahbar A, Miller M. Firearm Storage Practices Among American Veterans. Am J Prev Med. 2018 Aug 23. pii: S0749-3797(18)31697-0. doi: 10.1016/j.amepre.2018.04.014. [Epub ahead of print] PubMed PMID: 30166080. | <https://www.ncbi.nlm.nih.gov/pubmed/30166080> |
| 9 | Prescot AP, Prisciandaro JJ, Miller SR, Ingenito G, Kondo DG, Renshaw PF. Two-Dimensional Proton Magnetic Resonance Spectroscopy versus J-Editing for GABA Quantification in Human Brain: Insights from a GABA-Aminotransferase Inhibitor Study. Sci Rep. 2018 Sep 4;8(1):13200. doi: 10.1038/s41598-018-31591-3. PubMed PMID: 30181656. | <http://www.ncbi.nlm.nih.gov/pubmed/30181656> |
| 10 | Hoisington AJ, Billera DM, Bates KL, Stamper CE, Stearns-Yoder KA, Lowry CA, Brenner LA. Exploring service dogs for rehabilitation of veterans with PTSD: A microbiome perspective. Rehabil Psychol. 2018 Sep 13. doi: 10.1037/rep0000237. [Epub ahead of print] PubMed PMID: 30211604. | <http://www.ncbi.nlm.nih.gov/pubmed/30211604> |
| 11 | Barnes SM, Monteith LL, Forster JE, Nazem S, Borges LM, Stearns-Yoder KA, Bahraini NH. Developing Predictive Models to Enhance Clinician Prediction of Suicide Attempts Among Veterans With and Without PTSD. Suicide Life Threat Behav. 2018 Sep 11. doi: 10.1111/sltb.12511. [Epub ahead of print] PubMed PMID: 30206955. | <https://www.ncbi.nlm.nih.gov/pubmed/30206955> |
| 12 | Ferdosi H, Schwab KA, Metti A, Brenner LA, Terrio H, Pazdan RM, Cole WR, Scher AI. Trajectory of Postconcussive Symptoms 12-Months Post-Deployment in Soldiers with and without Mild Traumatic Brain Injury - Warrior STRONG Study. Am J Epidemiol. 2018 Sep 7. doi: 10.1093/aje/kwy199. [Epub ahead of print] PubMed PMID: 30203085. | <https://www.ncbi.nlm.nih.gov/pubmed/30203085> |
| 13 | Curtin K, Fleckenstein AE, Keeshin BR, Yurgelun-Todd DA, Renshaw PF, Smith KR, Hanson GR. Increased risk of diseases of the basal ganglia and cerebellum in patients with a history of attention-deficit/hyperactivity disorder. Neuropsychopharmacology. 2018 Dec;43(13):2548-2555. doi: 10.1038/s41386-018-0207-5. Epub 2018 Sep 12. PubMed PMID: 30209407; PubMed Central PMCID: PMC6224615. | <https://www.ncbi.nlm.nih.gov/pubmed/30209407> |
| 14 | Monteith LL, Bahraini NH, Gerber HR, Dorsey Holliman B, Schneider AL, Holliday R, Matarazzo BB. Military sexual trauma survivors' perceptions of veterans health administration care: A qualitative examination. Psychol Serv. 2018 Sep 27. doi: 10.1037/ser0000290. [Epub ahead of print] PubMed PMID: 30265071. | <https://www.ncbi.nlm.nih.gov/pubmed/30265071> |
| 15 | Corona CD, Gutierrez PM, Wagner BM, Jobes DA. The psychometric properties of the Collaborative Assessment and Management of Suicidality rating scale. J Clin Psychol. 2018 Oct 6. doi: 10.1002/jclp.22699. [Epub ahead of print] PubMed PMID: 30291761. | <https://www.ncbi.nlm.nih.gov/pubmed/30291761> |
| 16 | Zwir I, Arnedo J, Del-Val C, Pulkki-Råback L, Konte B, Yang SS, Romero-Zaliz R, Hintsanen M, Cloninger KM, Garcia D, Svrakic DM, Rozsa S, Martinez M, Lyytikäinen LP, Giegling I, Kähönen M, Hernandez-Cuervo H, Seppälä I, Raitoharju E, de Erausquin GA, Raitakari O, Rujescu D, Postolache TT, Sung J, Keltikangas-Järvinen L, Lehtimäki T, Cloninger CR. Uncovering the complex genetics of human temperament. Mol Psychiatry. 2018 Oct 2. doi: 10.1038/s41380-018-0264-5. [Epub ahead of print] PubMed PMID: 30279457. | <https://www.ncbi.nlm.nih.gov/pubmed/30279457> |
| 17 | Akram F, Fuchs D, Daue M, Nijjar G, Ryan A, Benros ME, Okusaga O, Baca-Garcia E, Brenner LA, Lowry CA, Ryan KA, Pavlovich M, Mitchell BD, Snitker S, Postolache TT. Association of plasma nitrite levels with obesity and metabolic syndrome in the Old Order Amish. Obes Sci Pract. 2018 Aug 1;4(5):468-476. doi: 10.1002/osp4.290. eCollection 2018 Oct. PubMed PMID: 30338117; PubMed Central PMCID: PMC6180710. | <https://www.ncbi.nlm.nih.gov/pubmed/30338117> |
| 18 | Nock MK, Han G, Millner AJ, Gutierrez PM, Joiner TE, Hwang I, King A, Naifeh JA, Sampson NA, Zaslavsky AM, Stein MB, Ursano RJ, Kessler RC. Patterns and predictors of persistence of suicide ideation: Results from the Army Study to Assess Risk and Resilience in Servicemembers (Army STARRS). J Abnorm Psychol. 2018 Oct;127(7):650-658. doi: 10.1037/abn0000379. PubMed PMID: 30335437. | <https://www.ncbi.nlm.nih.gov/pubmed/30335437> |
| 19 | Hom MA, Duffy ME, Rogers ML, Hanson JE, Gutierrez PM, Joiner TE. Examining the link between prior suicidality and subsequent suicidal ideation among high-risk US military service members. Psychol Med. 2018 Oct 25:1-10. doi: 10.1017/S0033291718003124. [Epub ahead of print] PubMed PMID: 30355371. | <https://www.ncbi.nlm.nih.gov/pubmed/30355371> |
| 20 | Matarazzo BB, Brown GK, Stanley B, Forster JE, Billera M, Currier GW, Ghahramanlou-Holloway M, Brenner LA. Predictive Validity of the Columbia-Suicide Severity Rating Scale among a Cohort of At-risk Veterans. Suicide Life Threat Behav. 2018 Oct 9. doi: 10.1111/sltb.12515. [Epub ahead of print] PubMed PMID: 30368871. | <https://www.ncbi.nlm.nih.gov/pubmed/30368871> |
| 21 | Zwir I, Arnedo J, Del-Val C, Pulkki-Råback L, Konte B, Yang SS, Romero-Zaliz R, Hintsanen M, Cloninger KM, Garcia D, Svrakic DM, Rozsa S, Martinez M, Lyytikäinen LP, Giegling I, Kähönen M, Hernandez-Cuervo H, Seppälä I, Raitoharju E, de Erausquin GA, Raitakari O, Rujescu D, Postolache TT, Sung J, Keltikangas-Järvinen L, Lehtimäki T, Cloninger CR. Uncovering the complex genetics of human character. Mol Psychiatry. 2018 Oct 3. doi: 10.1038/s41380-018-0263-6. [Epub ahead of print] PubMed PMID: 30283034. | <https://www.ncbi.nlm.nih.gov/pubmed/30283034> |
| 23 | Shura RD, Nazem S, Miskey HM, Hostetter TA, Rowland JA, Brenner LA, Va Mid-Atlantic Mirecc Workgroup, Taber KH. Relationship between traumatic brain injury history and recent suicidal ideation in Iraq/Afghanistan-era veterans. Psychol Serv. 2018 Nov 1. doi: 10.1037/ser0000208. [Epub ahead of print] PubMed PMID: 30382745. | <https://www.ncbi.nlm.nih.gov/pubmed/30382745> |
| 24 | Holder N, Holliday R, Suris A. The Effect of Childhood Sexual Assault History on Outpatient Cognitive Processing Therapy for Military Sexual Trauma-Related PTSD: A Preliminary Investigation. Stress Health. 2018 Sep 26. doi: 10.1002/smi.2838. [Epub ahead of print] PubMed PMID: 30259650. | <https://www.ncbi.nlm.nih.gov/pubmed/30259650> |
| 25 | Monteith LL, Hoffmire CA, Holliday R, Park CL, Mazure CM, Hoff RA. Do unit and post-deployment social support influence the association between deployment sexual trauma and suicidal ideation? Psychiatry Res. 2018 Oct 24;270:673-681. doi: 10.1016/j.psychres.2018.10.055. [Epub ahead of print] PubMed PMID: 30384288. | <https://www.ncbi.nlm.nih.gov/pubmed/30384288> |
| 26 | Stanley IH, Rogers ML, Hanson JE, Gutierrez PM, Joiner TE. PTSD symptom clusters and suicide attempts among high-risk military service members: A three-month prospective investigation. J Consult Clin Psychol. 2018 Nov 15. doi: 10.1037/ccp0000350. [Epub ahead of print] PubMed PMID: 30431299 | <https://www.ncbi.nlm.nih.gov/pubmed/30431299> |
| 27 | Brown TL, Gutierrez PM, Grunwald GK, DiGuiseppi C, Valuck RJ, Anderson HD. Access to Psychotropic Medication via Prescription Is Associated With Choice of Psychotropic Medication as Suicide Method: A Retrospective Study of 27,876 Suicide Attempts. J Clin Psychiatry. 2018 Nov 6;79(6). pii: 17m11982. doi: 10.4088/JCP.17m11982. PubMed PMID: 30418710. | <https://www.ncbi.nlm.nih.gov/pubmed/30418710> |
| 28 | Huber RS, Subramaniam P, Kondo DG, Shi X, Renshaw PF, Yurgelun-Todd DA. Reduced Lateral Orbitofrontal Cortex (OFC) Volume and Suicide Behavior in Youth with Bipolar Disorder. Bipolar Disord. 2018 Nov 24. doi: 10.1111/bdi.12729. [Epub ahead of print] PubMed PMID: 30471169. | <https://www.ncbi.nlm.nih.gov/pubmed/30471169> |
| 29 | Wortzel HS, Nazem S, Bahraini NH, Matarazzo BB, Silverman MM. The Potential Perils of a Suicide-specific Diagnosis. J Psychiatr Pract. 2018 Sep;24(5):354-358. doi: 10.1097/PRA.0000000000000333. PubMed PMID: 30427823 | <https://www.ncbi.nlm.nih.gov/pubmed/30427823> |
| 30 | Reger MA, Gebhardt HM, Lee JM, Ammerman BA, Tucker RP, Matarazzo BB, Wood AE, Ruskin DA. Veteran Preferences for the Caring Contacts Suicide Prevention Intervention. Suicide Life Threat Behav. 2018 Nov 19. doi: 10.1111/sltb.12528. [Epub ahead of print] PubMed PMID: 30451311. | <https://www.ncbi.nlm.nih.gov/pubmed/30451311> |
|  | Corona CD, Gutierrez PM, Wagner BM, Jobes DA. Assessing the Reliability of the CAMS Rating Scale Using a Generalizability Study. Crisis. 2018 Nov 26:1-7. doi: 10.1027/0227-5910/a000565. [Epub ahead of print] PubMed PMID: 30474407. | <https://www.ncbi.nlm.nih.gov/pubmed/30474407> |
| 31 | Brenner LA, Hoisington AJ, Stearns-Yoder KA, Stamper CE, Heinze JD, Postolache TT, Hadidi DA, Hoffmire CA, Stanislawski MA, Lowry CA. Military-Related Exposures, Social Determinants of Health, and Dysbiosis: The United States-Veteran Microbiome Project (US-VMP). Front Cell Infect Microbiol. 2018 Nov 19;8:400. doi: 10.3389/fcimb.2018.00400. eCollection 2018. PubMed PMID: 30510919; PubMed Central PMCID: PMC6252388. | <https://www.ncbi.nlm.nih.gov/pubmed/30510919> |
| 32 | Borges LM, Nazem S, Matarazzo BB, Barnes SM, Wortzel HS. Therapeutic Risk Management: Chain Analysis of Suicidal Ideation and Behavior. J Psychiatr Pract. 2019 Jan;25(1):46-53. doi: 10.1097/PRA.0000000000000358. PubMed PMID: 30633732. | <https://www.ncbi.nlm.nih.gov/pubmed/30633732> |
| 33 | Sheth C, Prescot AP, Legarreta M, Renshaw PF, McGlade E, Yurgelun-Todd D. Reduced gamma-amino butyric acid (GABA) and glutamine in the anterior cingulate cortex (ACC) of veterans exposed to trauma. J Affect Disord. 2019 Jan 31;248:166-174. doi: 10.1016/j.jad.2019.01.037. [Epub ahead of print] PubMed PMID: 30735853. | <https://www.ncbi.nlm.nih.gov/pubmed/30735853> |
| 34 | Postolache TT, Del Bosque-Plata L, Jabbour S, Vergare M, Wu R, Gragnoli C. Co-shared genetics and possible risk gene pathway partially explain the comorbidity of schizophrenia, major depressive disorder, type 2 diabetes, and metabolic syndrome. Am J Med Genet B Neuropsychiatr Genet. 2019 Feb 6. doi: 10.1002/ajmg.b.32712. [Epub ahead of print] Review. PubMed PMID: 30729689. | <https://www.ncbi.nlm.nih.gov/pubmed/30729689> |
| 35 | Currier JM, McDermott RC, Farnsworth JK, Borges LM. Temporal Associations Between Moral Injury and Posttraumatic Stress Disorder Symptom Clusters in Military Veterans. J Trauma Stress. 2019 Feb 4. doi: 10.1002/jts.22367. [Epub ahead of print] PubMed PMID: 30715755. | <https://www.ncbi.nlm.nih.gov/pubmed/30715755> |
| 36 | Holliday R, Monteith LL. Seeking help for the health sequelae of military sexual trauma: a theory-driven model of the role of institutional betrayal. J Trauma Dissociation. 2019 Feb 4:1-17. doi: 10.1080/15299732.2019.1571888. [Epub ahead of print] PubMed PMID: 30714879. | <https://www.ncbi.nlm.nih.gov/pubmed/30714879> |
| 37 | Keaton SA, Madaj ZB, Heilman P, Smart L, Grit J, Gibbons R, Postolache TT, Roaten K, Achtyes ED, Brundin L. An inflammatory profile linked to increased suicide risk. J Affect Disord. 2019 Jan 3;247:57-65. doi: 10.1016/j.jad.2018.12.100. [Epub ahead of print] PubMed PMID: 30654266. | <https://www.ncbi.nlm.nih.gov/pubmed/30654266> |
| 38 | Chen JI, Osman A, Freedenthal SL, Gutierrez PM. An Examination of the Psychometric Properties of the Reasons for Living Inventory within a Male Veteran Clinical Sample. Arch Suicide Res. 2019 Jan 13:1-50. doi: 10.1080/13811118.2018.1563576. [Epub ahead of print] PubMed PMID: 30636520 | <https://www.ncbi.nlm.nih.gov/pubmed/30636520> |
| 39 | Holliday R, Monteith LL, Wortzel HS. Understanding, Assessing, and Conceptualizing Suicide Risk Among Veterans With Posttraumatic Stress Disorder. Fed Pract. 2018 Apr;35(4):24-27. PubMed PMID: 30766350. | <https://www.ncbi.nlm.nih.gov/pubmed/30766350> |
| 40 | Gjervig Hansen H, Köhler-Forsberg O, Petersen L, Nordentoft M, Postolache TT, Erlangsen A, Benros ME. Infections, Anti-infective Agents, and Risk of Deliberate Self-harm and Suicide in a Young Cohort: A Nationwide Study. Biol Psychiatry. 2018 Nov 22. pii: S0006-3223(18)32020-1. doi: 10.1016/j.biopsych.2018.11.008. [Epub ahead of print] PubMed PMID: 30563760. | <https://www.ncbi.nlm.nih.gov/pubmed/30563760> |
| 41 | Brostow DP, Gunzburger E, Abbate LM, Brenner LA, Thomas KS. Mental Illness, Not Obesity Status, is Associated with Food Insecurity Among the Elderly in the Health and Retirement Study. J Nutr Gerontol Geriatr. 2019 Feb 22:1-24. doi: 10.1080/21551197.2019.1565901. [Epub ahead of print] PubMed PMID: 30794096. | <https://www.ncbi.nlm.nih.gov/pubmed/30794096> |
| 42 | Brostow DP, Warsavage TJ, Abbate LM, Starosta AJ, Brenner LA, Plomondon ME, Valle JA. Mental illness and obesity among Veterans undergoing percutaneous coronary intervention: Insights from the VA CART program. Clin Obes. 2019 Feb 21:e12300. doi: 10.1111/cob.12300. [Epub ahead of print] PubMed PMID: 30793500. | <https://www.ncbi.nlm.nih.gov/pubmed/30793500> |
| 43 | Makkar H, Reynolds MA, Wadhawan A, Dagdag A, Merchant AT, Postolache TT. Periodontal, metabolic, and cardiovascular disease: Exploring the role of inflammation and mental health. Pteridines. 2018 Feb;29(1):124-163. doi: 10.1515/pteridines-2018-0013. Epub 2018 Nov 13. PubMed PMID: 30705520; PubMed Central PMCID: PMC6350811. | <https://www.ncbi.nlm.nih.gov/pubmed/30705520> |
| 44 | Brenner LA, Bahraini NH. Concussion and risk of suicide: who, when and under what circumstances? Nat Rev Neurol. 2019 Jan 29. doi: 10.1038/s41582-019-0136-x. [Epub ahead of print] PubMed PMID: 30696951. | <https://www.ncbi.nlm.nih.gov/pubmed/30696951> |
| 45 | Bernecker SL, Zuromski KL, Gutierrez PM, Joiner TE, King AJ, Liu H, Nock MK, Sampson NA, Zaslavsky AM, Stein MB, Ursano RJ, Kessler RC. Predicting suicide attempts among soldiers who deny suicidal ideation in the Army Study to Assess Risk and Resilience in Servicemembers (Army STARRS). Behav Res Ther. 2018 Dec 6. pii: S0005-7967(18)30199-2. doi: 10.1016/j.brat.2018.11.018. [Epub ahead of print] PubMed PMID: 30598236. | <https://www.ncbi.nlm.nih.gov/pubmed/30598236> |
| 46 | Zuromski KL, Bernecker SL, Gutierrez PM, Joiner TE, King AJ, Liu H, Naifeh JA, Nock MK, Sampson NA, Zaslavsky AM, Stein MB, Ursano RJ, Kessler RC. Assessment of a Risk Index for Suicide Attempts Among US Army Soldiers With Suicide Ideation: Analysis of Data From the Army Study to Assess Risk and Resilience in Servicemembers (Army STARRS). JAMA Netw Open. 2019 Mar 1;2(3):e190766. doi: 10.1001/jamanetworkopen.2019.0766. PubMed PMID: 30874786. | <https://www.ncbi.nlm.nih.gov/pubmed/30874786> |
| 47 | Hoffmire CA, Monteith LL, Holliday R, Park CL, Brenner LA, Hoff RA. Administrative Military Discharge and Suicidal Ideation Among Post-9/11 Veterans. Am J Prev Med. 2019 Mar 18. pii: S0749-3797(19)30037-6. doi: 10.1016/j.amepre.2018.12.014. [Epub ahead of print] PubMed PMID: 30898536. | <https://www.ncbi.nlm.nih.gov/pubmed/30898536> |
| 48 | Corona CC, Zhang M, Wadhawan A, Daue ML, Groer MW, Dagdag A, Lowry CA, Hoisington AJ, Ryan KA, Stiller JW, Fuchs D, Mitchell BD, Postolache TT. Toxoplasma gondii IgG associations with sleep-wake problems, sleep duration and timing. Pteridines. 2019 Feb;30(1):1-9. doi: 10.1515/pteridines-2019-0001. Epub 2019 Feb 19. PubMed PMID: 30918422; PubMed Central PMCID: PMC6433149. | <https://www.ncbi.nlm.nih.gov/pubmed/30918422> |
| 49 | Wadhawan A, Stiller JW, Potocki E, Okusaga O, Dagdag A, Lowry CA, Benros ME, Postolache TT. Traumatic Brain Injury and Suicidal Behavior: A Review. J Alzheimers Dis. 2019 Mar 18. doi: 10.3233/JAD-181055. [Epub ahead of print] PubMed PMID: 30909230. | <https://www.ncbi.nlm.nih.gov/pubmed/30909230> |
| 50 | Andresen FJ, Monteith LL, Kugler J, Cruz RA, Blais RK. Institutional betrayal following military sexual trauma is associated with more severe depression and specific posttraumatic stress disorder symptom clusters. J Clin Psychol. 2019 Apr 4. doi: 10.1002/jclp.22773. [Epub ahead of print] PubMed PMID: 30947374. | <https://www.ncbi.nlm.nih.gov/pubmed/30947374> |
