# Supplementary material for: Beyond Journals—Visual Abstracts Promote Wider Suicide Prevention Research Dissemination and Engagement: A Randomized Crossover Trial
Source: Front Res Metr Anal. 2020 Oct 14;5:564193. doi: 10.3389/frma.2020.564193 (PMC8028397; doi:10.3389/frma.2020.564193)
Supplement: Supplementary file 3 [file Table_3.DOCX]

# Supplemental Table 3. Highest Performing Visual Abstracts

| **Visual Abstract Metric (n)** | **Sample Visual Abstract** |
| --- | --- |
| Most impressions (4,188) | 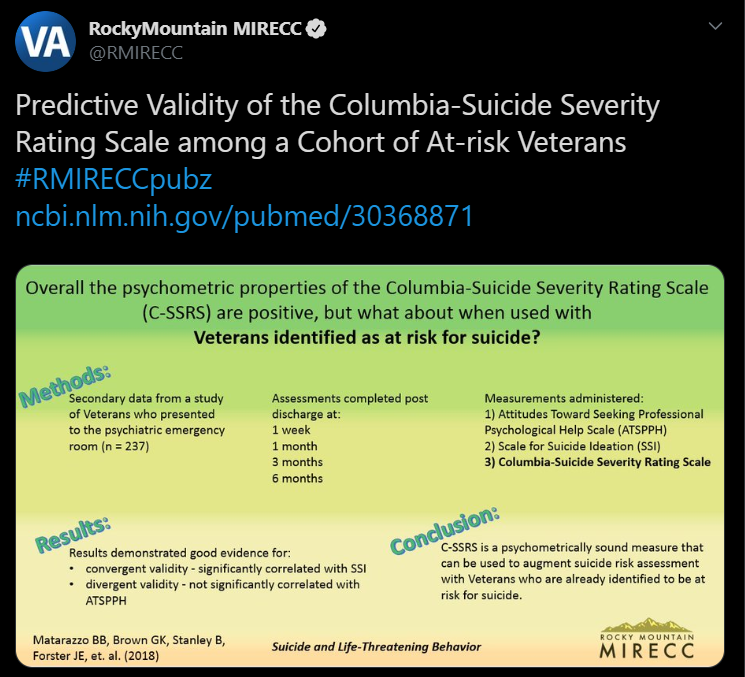 |
| Most retweets (14) | 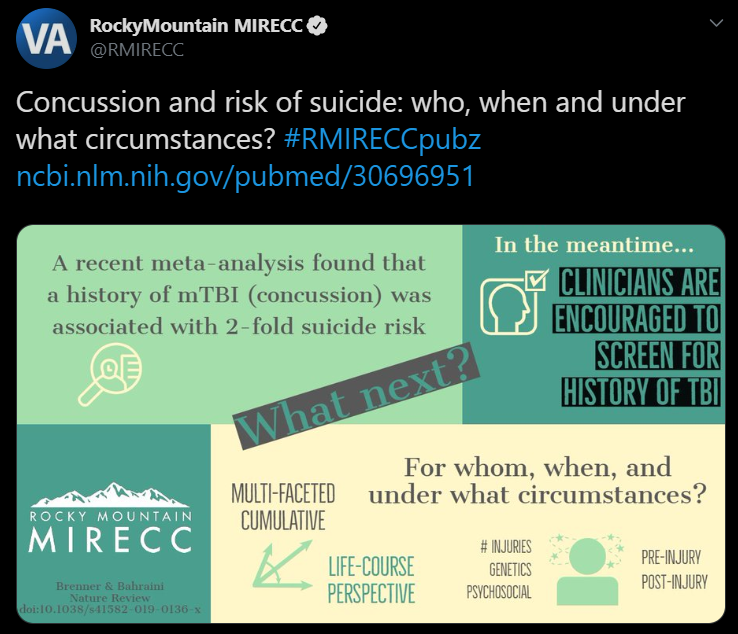 |
| Most link clicks (18) | 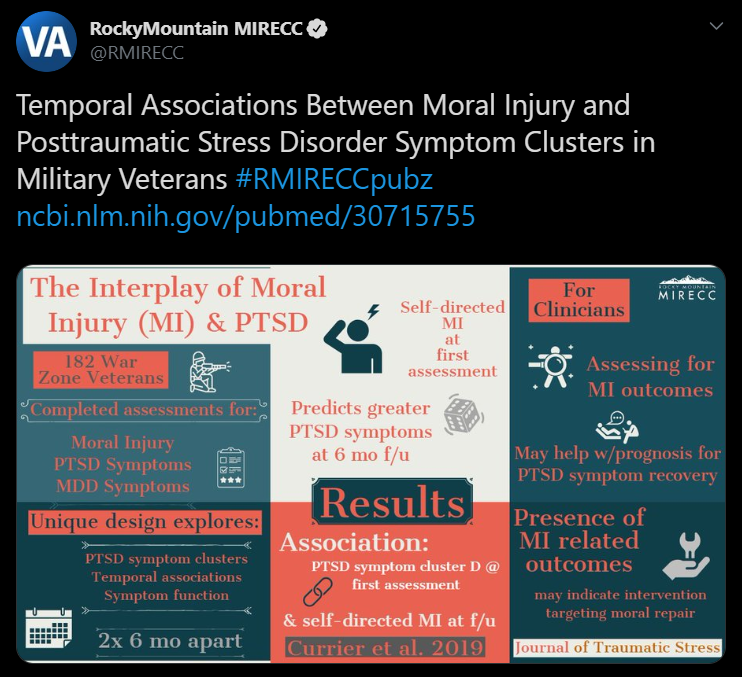 |
