# Supplementary material for: Beyond Journals—Visual Abstracts Promote Wider Suicide Prevention Research Dissemination and Engagement: A Randomized Crossover Trial
Source: Front Res Metr Anal. 2020 Oct 14;5:564193. doi: 10.3389/frma.2020.564193 (PMC8028397; doi:10.3389/frma.2020.564193)
Supplement: Supplementary file 4 [file Table_4.DOCX]

# Supplemental Table 4. Outcome Measurement

| **Assessment** | **Timing** | **Outcomes Measured** | **Data Source** |
| --- | --- | --- | --- |
| Time 1 | Baseline | Altmetrics | Altmetric It |
| Time 2 | 1 month | Impressions, retweets, link clicks, total engagements, Altmetrics | Twitter Analytics, Altmetric It |
| Time 3 | 2 months | Impressions, retweets, link clicks, total engagements, Altmetrics | Twitter Analytics, Altmetric It |
